# Evidence of the Anti-Inflammatory Effects of Probiotics and Synbiotics in Intestinal Chronic Diseases

**DOI:** 10.3390/nu9060555

**Published:** 2017-05-28

**Authors:** Julio Plaza-Díaz, Francisco Javier Ruiz-Ojeda, Laura Maria Vilchez-Padial, Angel Gil

**Affiliations:** 1 Department of Biochemistry and Molecular Biology II, School of Pharmacy, University of Granada, Granada 18071, Spain; jrplaza@ugr.es (J.P.-D.); fruizojeda@ugr.es (F.J.R.-O.); 2 Institute of Nutrition and Food Technology “José Mataix”, Biomedical Research Center, University of Granada, Armilla, Granada 18016, Spain; lauramvilchez@gmail.com; 3 Instituto de Investigación Biosanitaria ibs., GRANADA, Complejo Hospitalario Universitario de Granada, Granada 18014, Spain; 4 CIBEROBN (Physiopathology of Obesity and Nutrition CB12/03/30038), Instituto de Salud Carlos III (ISCIII), Madrid 28029, Spain

**Keywords:** probiotics, intestinal diseases, anti-inflammatory effects, inflammatory bowel diseases

## Abstract

Probiotics and synbiotics are used to treat chronic diseases, principally due to their role in immune system modulation and the anti-inflammatory response. The present study reviewed the effects of probiotics and synbiotics on intestinal chronic diseases in in vitro, animal, and human studies, particularly in randomized clinical trials. The selected probiotics exhibit in vitro anti-inflammatory properties. Probiotic strains and cell-free supernatants reduced the expression of pro-inflammatory cytokines via action that is principally mediated by toll-like receptors. Probiotic administration improved the clinical symptoms, histological alterations, and mucus production in most of the evaluated animal studies, but some results suggest that caution should be taken when administering these agents in the relapse stages of IBD. In addition, no effects on chronic enteropathies were reported. Probiotic supplementation appears to be potentially well tolerated, effective, and safe in patients with IBD, in both CD and UC. Indeed, probiotics such as *Bifidobacterium longum* 536 improved the clinical symptoms in patients with mild to moderate active UC. Although it has been proposed that probiotics can provide benefits in certain conditions, the risks and benefits should be carefully assessed before initiating any therapy in patients with IBD. For this reason, further studies are required to understand the precise mechanism by which probiotics and synbiotics affect these diseases.

## 1. Introduction

In 1907, the Russian Ilya Ilyich Mechnikov suggested that microbial ingestion improved host health. Indeed, he hypothesized that the consumption of lactic-acid-producing bacteria (LAB) strains found in yogurt might enhance longevity [1].

LAB is a heterogeneous group of microorganisms that are often present in a person’s gut, introduced through the ingestion of fermented foods, as well as in the gastrointestinal and urogenital tract of animals. Some of these strains have probiotic effects [2]. In particular, strains belonging to *Bifidobacterium, Enterococcus*, and *Lactobacillus* are the most widely used probiotic bacteria [3,4,5].

Werner Kollath was probably the first person to use the word "probiotic” in 1953 [6]. In current use, the term refers to microorganisms that confer a health benefit to the host when administered in adequate amounts [7,8]. In addition, dead bacteria and bacterial molecular components may exhibit probiotic properties [9]. In 2014, the International Scientific Association for Probiotics and Prebiotics stated that the development of metabolic by-products, dead microorganisms, or other microbial-based, nonviable products has potential; however, these do not fall under the probiotic construct [10].

When ingested, probiotics produce microbial transformation in the intestinal microbiota and exert several health-promoting properties, including maintenance of the gut barrier function and modulation of the host immune system [11,12,13,14,15,16].

By contrast, a prebiotic is a non-viable food component that confers a health benefit on the host that is associated with the modulation of the intestinal microbiota. Prebiotics may be a fiber, but a fiber is not necessarily a prebiotic. Using prebiotics and probiotics in combination is often described as synbiotics, but only if the net health benefit is synergistic [17,18].

Hence, probiotics and synbiotics are consumed in numerous and diverse forms, such as yogurt and fermented milks, cheese, and other fermented foods. The use of probiotics and synbiotics in preventive medicine to maintain a healthy intestinal function is well documented. In addition, both probiotics and synbiotics have been proposed as therapeutic agents for gastrointestinal disorders and other pathologies [19,20].

Intestinal diseases, particularly infectious illnesses, were first recognized as a major health issue in developing countries. However, intestinal chronic diseases are more prevalent in developed regions, and their incidence has continued to increase over the past several decades in various regions worldwide [21]. The exact pathogenic mechanism of the onset of selected intestinal chronic diseases remains mostly unexplained [22]. The principal clinical manifestations of intestinal chronic diseases are inflammatory bowel diseases (IBDs), necrotizing enterocolitis (NEC), and malabsorption syndromes. 

IBD is a term used to describe four pathologies: ulcerative colitis (UC), Crohn’s disease (CD), pouchitis, and microscopic colitis. These conditions are systemic disorders that affect the gastrointestinal tract and have frequent extraintestinal manifestations [23,24], in which the epithelial barrier function is a critical factor for onset. In addition, native immunity and commensal enteric bacteria play a role [25,26].

The current treatment of IBD first involves the induction of remission, which is followed by maintaining remission. Patients with an active disease are treated with topical or systemic 5-aminosalicylic acids (5-ASA), corticosteroids, or immunomodulators, such as azathioprine and 6-mercaptopurine, in addition to anti-TNF monoclonal antibodies [27,28].

Data from clinical trials indicate that certain intestinal disease conditions, including NEC, pouchitis, UC, and irritable bowel syndrome (IBS), have yielded clinical benefits with some probiotic and synbiotic interventions [29], because probiotics largely act directly or indirectly on the intestinal microbiota [30].

Several experimental methods are available to assess the effect of probiotics or synbiotics on intestinal diseases, especially their anti-inflammatory properties. Both in vitro and in vivo studies have been conducted. In vitro studies principally involve intestinal porcine epithelial cells (IPEC) J2, CaCo-2 cells, human dendritic cells (DC) obtained from peripheral blood and umbilical cord blood, monocyte-derived DCs, peripheral blood mononuclear cells, and intestinal T cells [31,32,33]. In vivo studies involve animal models (mice, rats, and dogs) with chemical inflammation induction or human patients. Despite this variety of studies, however, the mechanisms underlying the beneficial effects of probiotics or synbiotics remain incompletely understood.

Therefore, the present review was conducted to investigate the anti-inflammatory effects of probiotics and synbiotics on chronic intestinal diseases in in vitro and in vivo studies, as well as current evidence from human randomized clinical trials (RCTs).

## 2. Materials and Methods 

A comprehensive search of the relevant literature was performed using electronic databases, including MEDLINE (PubMed), EMBASE, and the Cochrane Library. MEDLINE through PubMed was searched for scientific articles that were published between 2010 and 2017 in English using the MeSH terms “probiotics” and “synbiotics”, combined with “intestinal diseases,” “Crohn’s disease,” and “ulcerative colitis”. We evaluated the results obtained using the following equation search: (“intestinal diseases”(All Fields) OR “Crohn’s disease”(All Fields) OR “colitis, Ulcerative”(All Fields)) and (“probiotics”(All Fields) OR “synbiotics”(All Fields)). Our search yielded 326 articles. A total of 38 articles were selected. Additionally, we searched the reference lists of the included articles for potential relevant literature.

## 3. Results

### 3.1. In Vitro Studies

Substantial evidence from in vitro studies suggests that known and potential probiotics exhibit strain-specific anti-inflammatory effects. A large inventory of animal and human cell lines is available as models of the gut [3], including DCs, porcine intestinal epitheliocyte (PIE) cells, intestinal epithelial cells (IEC)-6, HT-29, and IPEC-J2. In most of the in vitro experimental models, epithelial cells were cultivated as monolayers in which the establishment of a functional epithelial feature was not achieved.

DCs generate primary T-cell responses and mediate intestinal immune tolerance to prevent overt inflammation in response to gut microbiota. Indeed, DCs play a key role in UC pathogenesis [32,33]. *Lactobacillus casei* Shirota (LcS) was tested on human DCs from healthy controls and active UC patient samples. DCs from UC patients exhibit a reduced stimulatory capacity for the T-cell response and an enhanced expression of skin-homing markers, such as cutaneous lymphocyte-associated antigen (CLA) and C-C motif chemokine receptor 4 (CCR4) on stimulated T-cells. Those responses were characterized by increased interleukin (IL)-4 production and a loss of IL-22 and interferon (IFN)-γ secretion. LcS treatment restored the normal stimulatory capacity via a reduction in Toll-like receptor (TLR)-2 and TLR4 expression [32,33].

TLRs are transmembrane proteins expressed on various immune and non-immune cells, such as B-cells, natural killer cells, DCs, macrophages, fibroblast cells, epithelial cells, and endothelial cells. They are members of a family of evolutionarily conserved pattern recognition receptors that identify a wide range of microbial components [34].

*Lactobacillus plantarum* strain CGMCC1258 has a dual effect in an IPEC-J2 model that involves epithelial permeability, the expression of inflammatory cytokines, and an abundance of tight junction proteins. In this model, the damage was induced by enterotoxigenic *Escherichia coli* K88. The aforementioned probiotic strain decreased the transcript levels of IL-8, tumor necrosis factor (TNF-α), and negative regulators of TLRs, such as the single Ig Il-1-related receptor (SIGIRR), B-cell CLL/lymphoma 3 (Bcl3), and mitogen-activated protein kinase phosphatase-1 (MKP-1). Moreover, *L. plantarum* treatment reduced the gene and protein expression of occludin [35]. These results indicated that *L. plantarum* reduced the expression of pro-inflammatory cytokines induced by *E. coli* K88, possibly by modulating the TLR, nuclear factor kappa-B (NF-κB), and mitogen-activated protein kinase (MAPK) pathways [35].

The anti-inflammatory effects of *Lactobacillus delbrueckii* subsp. *delbrueckii* TUA4408L and its extracellular polysaccharide against *E. coli* 987P were evaluated in PIE cells. The activation of the MAPK and NF-κB pathways induced by *E. coli* 987P was downregulated via the upregulation of TLR negative regulators. In fact, TLR2 had a principal role in the immunomodulatory action of the probiotic strain [36].

Cell-free supernatants (CFS) from *E. coli* Nissle 1917 and *Lactobacillus rhamnosus* GG were evaluated for their capacity to prevent 5-fluorouracil-induced damage to IEC-6. Pre-treatment with the supernatants of those strains prevents or inhibits enterocyte apoptosis and the loss of the intestinal barrier function induced by 5-fluorouracil, potentially forming the basis of a preventative treatment modality for mucositis [37].

Finally, our investigation group assessed in vitro studies related to probiotics and their anti-inflammatory effects. Indeed, selected probiotics have been shown to modulate immune responses and inflammatory biomarkers in human DCs generated from CD34+ progenitor cells (hematopoietic stem cells) harvested from umbilical cord blood. The DCs exhibited surface antigens of dendritic Langerhans cells similar to the lamina propria DCs in the gut [38,39,40]. We co-incubated these intestinal-like human DCs with *Bifidobacterium breve* CNCM I-4035 or its CFS, *Salmonella typhi* CECT 725, or a combination of these treatments for 4 h. These treatments up-regulated TLR-9 gene transcription. In addition, CFS was a more powerful inducer of TLR-9 expression compared with probiotic bacteria in the presence of *S. typhi*. Both treatments induced Toll-interacting protein (TOLLIP) gene expression. Furthermore, CFS decreased the pro-inflammatory cytokines and chemokines in DCs that were challenged with *S. typhi*. By contrast, *B. breve* CNCM I-4035 was a potent inducer of the pro-inflammatory cytokines TNF-α, IL-8, and RANTES (regulated upon the activation of normal T cells, expressed, and presumably secreted), and anti-inflammatory cytokines, including IL-10. CFS restored the transforming growth factor (TGF)-β levels in the presence of *S. typhi*. These results indicate that *B. breve* CNCM I-4035 affects the intestinal immune response, whereas its supernatant exerts anti-inflammatory effects that are mediated by DCs. Similarly, *Lactobacillus paracasei* CNCM I-4034 and its CFS decreased pro-inflammatory cytokines and chemokines in human intestinal DCs that were challenged with *S. typhi* CECT 725. CFS was as effective as bacteria in reducing pro-inflammatory cytokine expression. These treatments strongly induced the transcription of the TLR-9 gene. In addition, an upregulation of the CASP8 and TOLLIP genes was observed. *L. paracasei* CNCM I-4034 was a potent inducer of TGF-β2 secretion, whereas the supernatant enhanced the innate immunity through the activation of TLR signaling. Moreover, *L. rhamnosus* CNCM I-4036 and its CFS were challenged with *E. coli* CECT 742, CECT 515, and CECT 729. *L. rhamnosus* treatment induced the production of TGF-β1 and TGF-β2, whereas the CFS increased TGF-β1 secretion. The two treatments induced the gene transcription of TLR-9. *L. rhamnosus* activated TLR-2 and TLR-4 gene expression, whereas CFS increased TLR-1 and TLR-5 gene expression [38,39,40].

Other selected probiotics exhibit in vitro anti-inflammatory properties. Both probiotic strains and CFS reduced the expression of pro-inflammatory cytokines via an action principally mediated by TLRs. Table 1 summarizes the principal investigations of probiotic effects in in vitro studies.

### 3.2. In Vivo Studies

#### 3.2.1. Animals

The anti-inflammatory effects of probiotics have been demonstrated in experimental models. Probiotic supplementation provides protective effects during spontaneous and chemically induced colitis by downregulating the production of inflammatory cytokines or by inducing regulatory mechanisms in a strain-specific manner [3].

##### Dextran Sulfate Sodium

The anti-inflammatory effects of *Lactobacillus acidophilus*, *L. plantarum*, *Bifidobacterium lactis*, *B. breve*, and inulin on UC colitis have been investigated. Acute UC was induced in Swiss mice using dextran sulfate sodium (DSS). The production of nitric oxide (NO) was evaluated in the supernatants of peritoneal macrophage cultures. The oral administration of probiotic strains and inulin reduced the severity of DSS-induced colitis. These treatments lead to a reduction in NO levels in peritoneal macrophage cultures [41]. The same mixture of strains was tested in another DSS-induced colitis animal model for seven days. Probiotic administration improved clinical symptoms and histological alterations observed in the colitis group, reduced NO production by peritoneal macrophages in DSS-treated mice, and enhanced mucus production in both DSS-treated and healthy mice [42].

*Lactobacillus reuteri* BR11 reduces the severity of experimental IBD, principally via a mechanism of thiol production [43]. Male Sprague–Dawley rats were administered 2% DSS to induce colitis. *L. reuteri* BR11 or *L. reuteri* BR11 mutants deficient in the cystine-uptake system were administered for 12 days. DSS administration resulted in significant colonic deterioration, including a reduced crypt area and increased damage severity. Probiotic administration partially alleviated the DSS effects, with a minor improvement in the crypt area. The administration of the mutant strain to colitic animals failed to produce significant differences when compared with the DSS control [43].

*Lactobacillus fermentum* CCTCC M206110, *Lactobacillus crispatus* CCTCC M206119, and *L. plantarum* NCIMB8826 were selected to assess the therapeutic effects on experimental colitis in BALB/c mice treated with DSS. *L. fermentum* CCTCC M206110 treatment resulted in reduced weight loss, colon length shortening, disease activity index scores, and histologic scores, whereas the *L. crispatus* CCTCC M206119 treatment group exhibited greater weight loss and colon length shortening, histologic scores, and more severe inflammatory infiltration. *L. plantarum* NCIMB8826 treatment improved the weight loss and colon length shortening, with no significant influence on the disease activity index and histologic damage in the colitis model [44]. The administration of an *L. crispatus* CCTCC M206119 supplement aggravated DSS-induced colitis, whereas *L. fermentum* CCTCC M206110 effectively attenuated DSS-induced colitis. The potential probiotic effect of *L. plantarum* NCIMB8826 on UC has not been assessed to date [44].

A previous study demonstrated that the intrarectal administration of mouse cathelin-related antimicrobial peptide (mCRAMP) alleviates DSS-induced colitis by preserving the mucus layer and reducing pro-inflammatory cytokines production [45]. A mutant of *Lactococcus lactis* NZ3900 that produces mCRAMP was tested in a murine model of DSS-induced colitis for seven days. Compared with the control group with colitis, cathelicidin-transformed *L. lactis* improved the clinical symptoms, maintained crypt integrity, and preserved the mucus content. The number of apoptotic cells, myeloperoxidase (MPO) activity, and malondialdehyde level were also significantly reduced. The increases in fecal microbiota in colitis animals were markedly prevented [45].

Hong et al., 2010 evaluated a mixture of *Lactobacillus brevis* HY7401, *Lactobacillus* sp. HY7801, and *Bifidobacterium longum* HY8004 in an acute DSS-induced colitis model for seven days [46]. Increased levels of acetate, butyrate, and glutamine, in addition to decreased levels of trimethylamine, were noted in the feces of the probiotic group compared with the DSS-alone-treated mice. The increased short chain fatty acid levels in the feces of mice fed the mixture indicates that probiotics have protective effects against DSS-induced colitis via modulation of the gut microbiota [46]. 

*E. coli* Nissle, 1917 was tested in a mouse model of reactivated colitis. Colitis was induced by adding DSS for five days. Two weeks later, colitis was reactivated by subsequent exposure to DSS. *E. coli* Nissle, 1917 administration exerted intestinal anti-inflammatory effects and attenuated colitis reactivation, as shown by reduced disease activity index values. Moreover, probiotic administration decreased the expression of pro-inflammatory cytokines and increased intestinal mucin-like and zona occludens-1 expression [47].

On the other hand, the effects of *L. rhamnosus* NutRes 1 and *B. breve* NutRes 204 on a DSS-induced chronic murine colitis model were assessed. Chronic colitis was induced by two DSS treatment cycles with a 10-day rest period. The probiotic supplementation was started after the first DSS treatment cycle and continued until the end of the experiment. *L. rhamnosus* NutRes 1, but not *B. breve* NutRes 204, rapidly and effectively improved the DSS-induced bloody diarrhea during the resolution phase. However, an increased expression of TLR2, TLR6, chemokine (C-C motif) ligand 2, IL-1β, TNF-α, and IL-6 was found in DSS-treated mice with *L. rhamnosus* supplementation. These results suggest caution in the use of probiotics in the relapse stages of IBD [48].

Capsules with bifidobacteria, lactobacilli, and *Streptococcus thermophilus* DSM24731 were administered to mice exposed to 5, 10, and 15 cycles of DSS. A probiotic mixture attenuated the disease activity index score and colon inflammation after 5, 10, and 15 cycles of DSS and reduced the histological alterations and the incidence of colonic dysplastic lesions in the three periods studied. In addition, the probiotic reduced the proliferating cell nuclear antigen labeling index and TNF-α, IL-1β, IL-6 production, and cyclooxygenase (COX)-2 expression, and increased IL-10 levels in colon tissue in the three periods assayed [49]. Additionally, in rats treated with DSS for seven days, the probiotic mixture exhibited anti-inflammatory properties, including reducing the disease activity index, MPO activity, iNOS, COX-2, NF-κB, TNF-α, IL-6, and p-Akt expression, and increasing IL-10 expression in colonic tissue. In addition, probiotic administration decreased TNF-α and IL-6, and increased IL-10 serum levels [50]. Moreover, probiotic administration was evaluated in acute intestinal ischemia/reperfusion injury in adult 129/SvEv mice. The mixture of strains reduced local tissue inflammation and injury. The reduction in local inflammation after a two-week course of the mixture was correlated with a significant reduction in active IL-1β levels and tissue levels of MPO. Active NF-κB levels were significantly higher in the control group, consistent with the tissue inflammation. Inflammation was attenuated by probiotic administration. Finally, the administration of bifidobacteria, lactobacilli, and *S. thermophilus* did not cause any systemic inflammation or lung injury [51].

*Bacillus subtilis* R179 also exhibits a protective effect in IBD. The effects of *B. subtilis* were analyzed using a mouse DSS model of colitis in which a higher dose ameliorated gut inflammation and dysbiosis [52].

##### 2,4,6-Trinitrobenzenesulfonic Acid

The impact of *L. plantarum* 21 on inflammatory mediators in 2,4,6-trinitrobenzenesulfonic acid (TNBS)-induced colitis in rats has been evaluated. Treatment with *L. plantarum* 21 for 14 days after the induction of colitis decreased thiobarbituric acid reactive substances (TBARS) and NO, and increased glutathione concentrations. The IL-1β and TNF-α proteins, in addition to mRNA expression, were down-regulated, whereas IL-10 protein and mRNA expression was up-regulated in *L. plantarum* 21-treated rats. In addition, probiotic treatment attenuated macroscopic colonic damage and histopathological changes produced by TNBS [53].

The effects of lactobacilli and bifidobacteria administration on TNF-α and TLR4 expression in a rat colitis model induced by TNBS were also investigated. No significant differences were found in TLR4 and TNF-α expression between the two-week probiotics treatment group and the colitis group, whereas significant reductions were found in rats treated with probiotics for four weeks compared with the TNBS group [54].

The effects of *Butyricicoccus pullicaecorum* CCUG 55,265 in a rat colitis model with TNBS and a Caco-2 cell model were analyzed. *B. pullicaecorum* administration resulted in a significant protective effect based on macroscopic and histological criteria and decreased intestinal MPO, TNF-α, and IL-12 levels. *B. pullicaecorum* supernatant prevented the increase in IL-8 secretion induced by TNF-α and IFN-γ in a Caco-2 cell model [55].

##### Other Intestinal Inflammation Models

Chronic enteropathies (CE) are believed to be caused by an aberrant immune response towards the intestinal microbiome. The in vitro effects of the probiotic *Enterococcus faecium* NCIMB 10,415 E1707 were previously evaluated using canine cells (e.g., whole blood, intestinal biopsies), but data on in vivo efficacy are lacking. Dogs diagnosed with CE were prospectively recruited to receive a hydrolyzed elimination diet, in addition to either a synbiotic product containing *E. faecium* NCIMB 10,415 E1707 or a placebo for six weeks. Both veterinary staff and owners were blinded to the treatment. Of the 45 cases recruited, 12 completed the clinical trial. Seven dogs received the symbiotic and five received the placebo product. No difference was noted between groups or treatments regarding the clinical efficacy and histology scores [56]. Casp-1 and NLRP3 gene expression was reduced in the CE samples when compared with the controls. Ex vivo treatment with *E. faecium* NCIMB 10,415 E1707 reduced NLRP3 expression in the control samples [57].

The effects of *L. delbrueckii* subsp. *bulgaricus* in an intestinal malfunction mouse model induced by lincomycin hydrochloride were tested. Consequently, *L. delbrueckii* administration increased secretory immunoglobulin A and decreased intestinal pathological damage [58].

*L. plantarum* LS/07 CCM7766 alone or in combination with inulin was assessed in rats with chronic inflammation. *N,N*-dimethylhydrazine administration triggered the production of IL-2, IL-6, IL-17, and TNF-α, as well as the expression of NF-κB, COX-2, and iNOS, and caused the depletion of goblet cells. *L. plantarum* LS/07 CCM7766 alone and in combination with inulin abolished the inflammatory process in the jejunal mucosa by inhibiting the production of pro-inflammatory cytokines and stimulating IL-10 cytokine synthesis, whereas the TGF-β1 levels did not change significantly [59].

Ogita et al., 2015 tested the effects of *L. rhamnosus* OLL2838, *Bifidobacterium infantis* ATCC 15,697, and *S. thermophilus* Sfi 39 on the maturation of bone marrow-derived DCs from mice [60]. *L. rhamnosus* OLL2838 induced appreciable levels of IL-10 and NO production, whereas *S. thermophilus* Sfi 39 essentially elicited IL-12 and TNF-α [60]. In addition, *L. rhamnosus* OLL2838 was evaluated in an in vivo model of gluten-specific enteropathy characterized by villus blunting, crypt hyperplasia, high levels of intestinal IFN-γ, increased cell apoptosisin lamina propria, and reduced intestinal glutathione S-transferase activity. Probiotic administration enhanced the total glutathione and glutathione S-transferase activity, whereas caspase-3 activity was reduced. However, the probiotic strain failed to recover the normal histology and further increased intestinal IFN-γ [60].

Finally, Wu et al. identified a novel role of probiotics in activating vitamin D receptor (VDR), thus inhibiting inflammation, using cell models and VDR knockout mice [61]. The probiotics *L. rhamnosus* GG ATCC 53,103 and *L. plantarum* increased VDR protein expression in both mouse and human intestinal epithelial cells. Moreover, the role of probiotics in regulating VDR signaling was assessed in vivo using a *Salmonella typhimurium* ATCC 14,028-induced colitis model in VDR knockout mice. Probiotic treatment conferred physiological and histologic protection from colitis in mice, whereas probiotics did not affect the knockout mice. Probiotic treatment also enhanced the number of Paneth cells, which secrete AMPs for host defense [61].

Animal studies seem to be more extensively used than cell models in the evaluation of probiotic properties. Probiotic administration might improve clinical symptoms, histological alterations, and mucus production in the majority of the evaluated studies, but some results suggest that caution should be taken when administering these agents in the relapse stages of IBD. In addition, no effects on chronic enteropathies were noted. Table 2 shows the main investigations regarding the probiotic anti-inflammatory effects in animal studies.

#### 3.2.2. Humans

##### Ulcerative Colitis

UC is a chronic IBD of unknown etiology that is characterized by acute exacerbations of intestinal complications, followed by remissions. Additionally, one of the main hypotheses is that UC is caused by an excessive immune response to endogenous bacteria in genetically predisposed individuals. Therefore, the manipulation of the mucosal microbiota to reduce the inflammatory potential of colonizing bacteria is an attractive therapy for UC. Recently, probiotic therapy has been demonstrated to be potentially effective and safe in patients with UC. Tamaki et al., 2016 investigated the efficacy and safety of probiotic treatment with *B. longum* 536, which is a probiotic isolated in 1969 from the feces of a breast-fed infant, in Japanese patients with active UC using an RCT [62]. The probiotic improved clinical symptoms, such as the UC disease activity index and Rachmilewitz endoscopic index, in patients with mild to moderately active UC, but further studies are needed to clarify the efficacy and safety of *B. longum* 536 for UC [62].

A single-center, randomized, double-blind, placebo-controlled study was conducted to examine whether 12 months of probiotic therapy, including a mixture of the strains *Streptococcus faecalis* T-110, *Clostridium butyricum* TO-A, and *Bacillus mesentericus* TO-A, was useful for preventing the relapse of UC in patients who were already in remission. The relapse rates in the probiotic therapy group increased significantly at three and nine months. However, no differences were noted at 12 months. Moreover, in a cluster analysis of fecal microbiota, which is a molecular technique that compares the diversity and colony structure of microbial complexes, seven patients belonged to cluster I; 32 to cluster II, which is the “appropriate intestinal microbiota”; and seven to cluster III. Therefore, probiotics may be effective for UC, especially in cluster I patients [63].

With regards to UC, a RCT was conducted to assess the clinical efficacy of profermin, a food with fermented oats containing *L. plantarum* 299v and other ingredients such as barley malt and lecithin, in relapsing UC. The patients with a mild-to-moderate flare-up of UC, which was defined as a Simple Clinical Colitis Activity Index (SCCAI) score ≥5 and ≤1, showed a significant decrease in the SCCAI score after probiotic supplementation. Thus, *L. plantarum* 299v administration was safe, well tolerated, and palatable, and induced a clinically significant reduction in the SCCAI score compared with a placebo in patients with mild-to-moderate flare-up of UC [64].

A recent case report of bacteremia was caused by *L. rhamnosus* GG in an adult patient affected by severe active UC under treatment with corticosteroids and mesalazine. *Lactobacillus* species are ubiquitous Gram-positive commensals of the normal human microbiota, but their role as opportunistic pathogens is emerging. The case of bacteremia was apparently associated with the translocation of bacteria and fungi from the intestinal lumen to the blood. Thus, *Candida* infection was likely promoted by previous extensive antibiotic use, whereas *L. rhamnosus* GG infection was most likely associated with the probiotic strain administered to the patient and possibly favored by the use of vancomycin, to which the strain was resistant. Notably, this observation based on pending conclusive evidence suggests that the use of probiotics should be considered with caution in cases of active severe IBD with mucosal disruption [65].

UC is also associated with fecal dysbiosis, and different human and animal studies suggest that the gastrointestinal microbiome may trigger the intestinal immune response. Fecal microbial transplantation (FMT) may be a therapeutic option. However, a clinical report published by Suskind et al. (2015) described that single-dose FMT via a nasogastric tube was well tolerated in four patients with UC, but no clinical benefit was demonstrated [66]. Recently, Paramsothy et al., 2017 have reported that intensive-dosing, multidonor FMT induces clinical remission and endoscopic improvement in active UC and is associated with distinct microbial changes that relate to the outcome [67].

Conversely, a 2014 case report by Brace et al. indicated that FMT may be a promising therapy for *Clostridium difficile* infection (CDI) in patients with IBD, because colonization with toxigenic *C. difficile* is significantly higher in IBD patients compared with the general population [68]. Thus, an IBD patient with two CDIs was treated 18 months apart, and each infection was successfully treated with FMT with no IBD flares or complications. Only this study described sequential FMT from a single donor for an IBD patient with CDI recurrences. Microbiota composition analysis indicated that the patient’s pre-transplant samples exhibited reduced diversity, with deficiencies in the usually dominant populations of *Firmicutes* and *Bacteroidetes*. This microbiota pattern is consistent with microbial analyses of non-IBD patients during CDI, thus supporting the theory that predominating *Firmicutes* and *Bacteroidetes* groups may confer colonization resistance against *C. difficile*. The authors described only one patient-donor set, which is the main limitation of this study; therefore, further studies are needed to approach the vulnerability profile of patients at risk of relapse [68].

A case report described by Vahabnezhad et al., 2013 reported that probiotic strains of *Lactobacillus*, *L. rhamnosus* GG, caused bacteremia in a 17-year-old boy with UC managed with systemic corticosteroids and infliximab, which is a tumor necrosis factor-α antagonist [69]. *L. rhamnosus* GG was assessed via blood culture on day 2, but the subsequent blood cultures on day 3 and 5 were negative. The patient was treated with antibiotics for five days and defervesced by day eight of his illness. The authors hypothesize that the immunosuppressive effects from systemic corticosteroids and infliximab may have also predisposed the patient to a higher risk of infection.

The risk of infection due to lactobacilli and bifidobacteria is extremely rare and represents 0.05–0.4% of cases of infective endocarditis and bacteremia. Meanwhile, historically, *Lactobacillus* spp. found in food has been considered to be insignificant and they are often regarded as contaminants when isolated from patient samples. Therefore, although probiotics can offer potential benefits in certain healthy conditions, their risks and benefits should still be carefully assessed in patients with some complications, especially when the patients might be immunocompromised [69,70].

##### Crohn’s Disease

CD is a systemic disorder in which the development of host genetic susceptibility represents an important etiological factor. Multiple studies have observed differences in the microbiotas of individuals with CD, with a reduction in anti-inflammatory bacteria and an increase in pro-inflammatory bacteria compared with the microbiotas of healthy subjects. In this sense, different studies have focused on reporting the effect of some strains in CD in recent years. Petersen et al., 2014 investigated the effects of *E. coli*, which is a member of the phylogenetic group B2, and its association with both CD and UC [71]. Interestingly, the probiotic *E. coli* Nissle, 1917 has an equivalent effect to mesalazine in preventing disease flares in UC patients. Moreover, antibiotics seem to have some effect in the treatment of IBD patients. Thus, these authors designed a study to investigate whether ciprofloxacin for one week, followed by therapy with *E. coli* Nissle, 1917 for seven weeks, or either of these treatments alone, influence the remission rate among UC patients. However, no benefit in the use of *E. coli* Nissle 1917 as an add-on treatment to conventional therapies for active UC was noted [71].

Fedorak et al., 2015 investigated the effects of capsules with bifidobacteria, lactobacilli, and *S. thermophilus* DSM24731 in preventing the recurrence of CD after surgery [72]. The recurrence after intestinal resection in CD is quite common. Thus, in this study, within 30 days of ileocolonic resection and re-anastomosis, patients with CD were randomly assigned to groups administered capsules versus a placebo. Although there were no differences in the endoscopic recurrence rates at day 90 between patients who received the probiotics strains, the mucosal levels of inflammatory cytokines, such as IL-8 and IL-1β, were lower among patients who received the probiotics. However, the authors concluded that additional studies are necessary to confirm the effect of the probiotic mixture in the prevention of postoperative recurrence [72].

Hevia et al., 2014 explored the levels of antibodies (IgG and IgA) raised against extracellular proteins produced by LAB and its association with IBD [73]. The presence of serum antibodies, such as IgG and IgA produced by food bacteria from the genera *Bifidobacterium* and *Lactobacillus,* which are used as serum biomarkers of CD or UC, were determined by western blot and ELISA in sera collections from healthy individuals, CD patients, and UC patients. The levels of IgA antibodies against a cell-wall hydrolase from *L. casei* subsp. *rhamnosus* GG (CWH) were significantly higher in the IBD group and appeared to have different immune responses to food bacteria. Specifically, IgA antibodies developed against an extracellular protein of *L. casei* are associated with IBD. Therefore, these results suggest that anti-CWH IgA levels have a potential use for the early detection of CD and UC. However, studies with larger sample sizes are necessary. In addition, the identification of other extracellular protein targets present in food and probiotic bacteria is needed [73].

Ahmed et al., 2013 performed a pilot study in patients with colitis, randomized to either receive a synbiotics for a month and then “crossed over” to receive a placebo or alternatively to receive the placebo first followed by the symbiotic [74]. The main results showed that there were no differences in colonic microbiota between patients with CD or UC, and the spectrum bacteria were not altered by synbiotic administration [74].

Pouchitis is a nonspecific inflammation of the pouch that is present in UC patients and remains the most common post-operative long-term complication. Inflammation of the pouch is characterized by increased stool frequency, rectal bleeding, abdominal cramping, urgency, and fever, and the pathogenesis of pouchitis is still poorly understood. However, there is evidence that implicates the gut microbiota, suggesting that probiotics could reduce the risk of the recurrence of pouchitis, but the mechanisms are not fully understood. Persborn et al., 2013 reported that treatment with probiotic mixture for eight weeks after antibiotics restored the increased permeation to *E. coli* K12 in 16 patients with chronic pouchitis [75]. Thirteen individuals served as a control. This finding could be an important factor for the prevention of recurrence during maintenance treatment with probiotics for this inflammatory status [75].

The bacteria *B. infantis* 35,624 exerts beneficial immunoregulatory effects by mimicking commensal-immune interactions. In an RCT, *B. infantis* 35,624 was used to assess the impact of oral administration for six to eight weeks on inflammatory biomarker and plasma cytokine levels in patients with UC, chronic fatigue syndrome, and psoriasis. *B. infantis* 35,624 reduced plasma CRP levels in all three inflammatory disorders compared with the placebo. Interestingly, plasma IL-6 was reduced in UC patients and chronic fatigue syndrome. Furthermore, in healthy subjects, LPS-stimulated TNF-α and IL-6 secretion by peripheral blood mononuclear cells was significantly reduced in the *B. infantis* 35,624-treated groups compared with the placebo following eight weeks of feeding. These findings demonstrate the reduction of systemic pro-inflammatory biomarkers by *B. infantis* 35,624 and the immunomodulatory effects of the microbiota in humans [76].

In humans, an RCT was described by Bourreille et al., 2013, which was a prospective study with 165 patients with CD who achieved remission after treatment with steroids or salicylates [77]. The patients were randomly assigned to groups that received *S. boulardii* or placebo for 52 weeks. No differences in the median time to relapse were noted between the groups. Thus, although the probiotic yeast *S. boulardii* effects were positive in animals, it does not appear to have any beneficial effects for patients with CD in remission after steroid or salicylate therapies [77]. 

In summary, the supplementation of selected probiotics appears to be potentially well tolerated, effective, and safe in patients with IBD, both CD and UC. Indeed, probiotics such as *B. longum* 536 improved clinical symptoms in patients with mild to moderately active UC. Although it has been proposed that probiotics can provide benefits in certain conditions, such as healthy individuals or individuals with obesity or metabolic syndrome, the risks and benefits should be carefully assessed before initiating any therapy in patients with IBD. Table 3 summarizes the studies of probiotics and synbiotics in humans.

## 4. Further Research and Directions

For a selected number of probiotics and synbiotics, the intestinal anti-inflammatory effects have been documented using in vitro and experimental approaches, as well as human studies. However, the appropriate doses and time of treatment have not been defined, and for a majority of them, the molecular mechanism of action has not been ascertained. Further research should evaluate the most appropriate doses and time of treatment for each probiotic and synbiotic in terms of efficacy in the control of specific chronic inflammatory diseases, and how they contribute to ameliorate the symptoms of the disease as related to the decrease of intestinal and systemic biomarkers of inflammation. In addition, the interactions of particular probiotics with cell receptors and how cell signaling cascades are affected, as well as how the expression of intestinal host genes involved in the immune and inflammatory responses are modulated, are key aspects to understand the action of probiotics. Moreover, the next generation sequence systems should contribute to knowledge on the individual intestinal ecology of patients affected with inflammatory intestinal diseases and the commensal and probiotics strains that actually have a key role in the control of biodiversity in the intestine. This should include the sequencing not only of the bacterial metagenomes, but also the intestinal viromes, thus envisaging the potentially efficient and safe fecal transplantation of healthy subjects to intestinal chronic inflamed patients.

**Figure 1:**
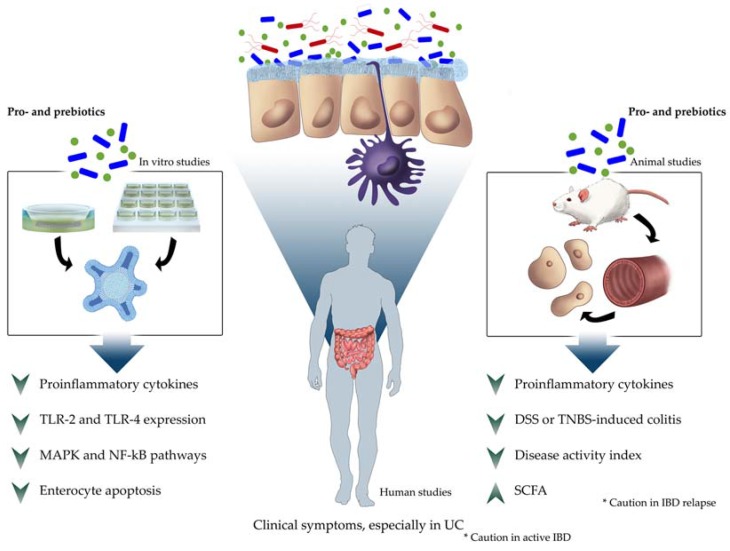
Summary of probiotic anti-inflammatory effects in intestinal chronic diseases in different scientific approaches. DSS, dextran sulfate sodium; IBD, inflammatory bowel disease; MAPK, mitogen-activated protein kinase; NF-κB, nuclear factor kappa-B; SCFA, short-chain fatty acids; TNBS, 2,4,6 trinitrobenzenesulfonic acid; TLR, toll-like receptor; UC, ulcerative colitis.

Finally, Figure 1 represents the summary of anti-inflammatory effects of probiotics and synbiotics in intestinal chronic diseases.

## Figures and Tables

**Table 1:** Summary of probiotic anti-inflammatory effects in In Vitro studies.

Reference	Cell Type	Probiotic Strain	Type of Study	Main Outcome
Mann et al. 2014, 2013 [32,33]	human DC	*L. casei* Shirota	In vitro	DC from UC patients samples have an increase of IL-4 production and loss of IL-22 and IFN-γ secretion. *L. casei* Shirota treatment restored the normal stimulatory capacity through a reduction in the TLR-2 and TLR4 expression
Wu et al., 2016 [35]	IPEC-J2 model	*L. plantarum* strain CGMCC1258	In vitro	*L. plantarum* decreased transcript abundances of IL-8, TNF-α, and negative regulators of TLRs. Moreover, *L. plantarum* treatment decreased the gene and protein expression of occludin
Wachi et al., 2014 [36]	PIE cells	*L. delbrueckii* subsp. *delbrueckii* TUA4408L	In vitro	The activation of MAPK and NF-κB pathways induced by *E. coli* 987P were downregulated through upregulation of TLR negative regulators, principally by TLR2
Prisciandaro et al., 2012 [37]	IEC-6	*E. coli* Nissle 1917 and *L. rhamnosus* GG	In vitro	Pre-treatment with these probiotics could prevent or inhibit enterocyte apoptosis and loss of intestinal barrier function induced by 5-FU
Bermudez-Brito et al., 2012, 2013, 2014 [13,38,39]	DC	*L. paracasei* CNCM I-4034, *B. breve* CNCM I-4035, and *L. rhamnosus* CNCM I-4036	In vitro	Induction of TLR-9 expression and TGF-β2 secretion. CFS treatment decreased the pro-inflammatory cytokines and chemokines

CFS, cell-free supernatant; DC, dendritic cells; FU, fluorouracil; IEC, intestinal epithelial cells; IL, interleukin; IFN, interferon; IPEC, intestinal porcine epithelial cells, MAPK, mitogen-activated protein kinase; NF-κB, nuclear factor κ-B; TGF, transforming growth factor; TNF-α, tumor factor necrosis alpha; TLR, toll-like receptor; UC, ulcerative colitis.

**Table 2:** Summary of probiotic anti-inflammatory effects in animal studies.

Reference	Animal Species	Probiotic Strain/Treatment	Type of Study	Main Outcome	Adverse Event/Adverse Effects
Abdelouhab et al., 2012 [41]	Swiss mice	*L. acidophilus, L. plantarum, B. lactis, B. breve,* and inulin	In vivo, DSS-induced colitis	Oral administration of probiotic strains and inulin decreased severity colitis	-
Toumi et al., 2013 [42]	Swiss mice	*L. acidophilus, L. plantarum, B. lactis, B. breve*	In vivo, DSS-induced colitis	Probiotic administration improved clinical symptoms, histological alterations, and mucus production	-
Atkins et al., 2012 [43]	Male Sprague–Dawley rats	*L. reuteri* BR11	In vivo, DSS-induced colitis	Probiotic administration partially alleviated the DSS effects, with a minor improvement in crypt area	-
Cui et al., 2016 [44]	BALB/c mice	*L. fermentum* CCTCC M206110, *L. crispatus* CCTCC M206119, and *L. plantarum* NCIMB8826	In vivo, DSS-induced colitis	*L. fermentum* CCTCC M206110 proved to be effective at attenuating DSS-induced colitis. Administration of *L. crispatus* CCTCC M206119 aggravated DSS-induced colitis	Administration of *L. crispatus* CCTCC M206119 aggravated DSS-induced colitis
Wong et al., 2012 [45]	BALB/c mice	A mutant of *L. lactis*	In vivo, DSS-induced colitis	*L. lactis* could improve the clinical symptoms, maintain crypt integrity and preserve mucus content. The number of apoptotic cells, MPO activity and malondialdehyde level were also significantly reduced	-
Zhang et al., 2016 [52]	Male C57 mice	*B. subtilis*	In vivo, DSS-induced colitis	*B. subtilis* treatment ameliorated gut inflammation and dysbiosis	-
Hong et al., 2010 [46]	Male ICR mice	*L. brevis* HY7401, *L.* sp. HY7801 and *B. longum* HY8004	In vivo, DSS-induced colitis	Increased levels of acetate, butyrate, and glutamine and decreased levels of trimethylamine	-
Garrido-Mesa et al., 2011 [47]	C57BL/6J mice	*E. coli* Nissle 1917	In vivo, DSS-induced colitis	*E. coli* Nissle 1917 administration exerted intestinal anti-inflammatory effect and attenuated the reactivation of the colitis	-
Zheng et al., 2016 [48]	Female C57BL/6 mice	*L. rhamnosus* NutRes 1 and *B. breve* NutRes 204	In vivo, DSS-induced colitis	An increased expression of inflammation markers were found in DSS-treated mice with *L. rhamnosus* supplementation	-
Talero et al., 2015 [49]	Female C57BL/6 mice	Capsules with bifidobacteria, lactobacilli, and *S. thermophilus*	In vivo, DSS-induced colitis	Probiotic mixture attenuated the disease activity index score and colon inflammation and also inflammation markers	-
Dai et al., 2013 [50]	Male Wistar rats	Capsules with bifidobacteria, lactobacilli, and *S. thermophilus*	In vivo, DSS-induced colitis	The probiotic mixture have anti-inflammatory properties reducing the disease activity index, MPO activity, inflammation biomarkers, and also increasing of IL-10 expression	-
Salim et al., 2013 [51]	Adult male 129/SvEv mice	Capsules with bifidobacteria, lactobacilli, and *S. thermophilus*	In vivo, acute intestinal ischemia/reperfusion injury	Levels of active NF-κB were significantly higher in the control group, corroborating with the inflammation of the tissue, which was attenuated by probiotic administration	-
Satish Kumar et al., 2015 [53]	Wistar female rats	*L. plantarum* 21	In vivo, TNBS-induced colitis	Treatment with *L. plantarum* 21 for 14 days after induction of colitis decreased TBARS, NO, IL-1β and TNF-α and increased glutathione concentration and IL-10 expression	-
Yang et al., 2013 [54]	Sprague-Dawley Rats	Lactobacilli and bifidobacteria	In vivo, TNBS-induced colitis	TLR4 and TNF-α expression were reduced with probiotics	-
Eeckhaut et al., 2013 [55]	Male Wistar rats	*B. pullicaecorum*	In vivo, TNBS-induced colitis	*B. pullicaecorum* administration resulted in a decreased intestinal MPO, TNF-α and IL-12 levels	-
Schmitz et al., 2015 [56,57]	Dogs	*E. faecium* NCIMB 10415 E1707	Chronic enteropathies	There was no difference between groups or treatments regarding clinical efficacy, histology scores	-
Sun et al., 2015 [58]	BALB/c mice	*L. delbrueckii*	Intestinal malfunction induced by Lincomycin hydrochloride	*L. delbrueckii* administration increased secretory immunoglobulin A and decreased the intestine pathological damage	-
Štofilová et al., 2015 [59]	Female Sprague Dawley rats	*L. plantarum* LS/07 CCM7766	In vivo, *N,N*-dimethylhydrazine-induced colitis	*L. plantarum* LS/07 CCM7766 and its combination with inulin abolished inflammatory process in the jejunal mucosa	-
Ogita et al., 2015 [60]	DQ8 transgenic mice	*L. rhamnosus* OLL2838*, B. infantis* ATCC 15697, and *S. thermophilus* Sfi 39	In vivo, model of gluten-specific enteropathy	Probiotic administration enhanced total glutathione and glutathione *S*-transferase activity, whereas caspase-3 activity was reduced	-
Wu et al., 2015 [61]	Female C57BL/6 mice	*L. rhamnosus* GG and *L. plantarum*	In vivo, vitamin D receptor knockout mice	Probiotic treatment conferred physiological and histologic protection from colitis	-

AE, adverse event; DSS, dextran sulfate sodium; IL, interleukin; MPO, myeloperoxidase; NF-κB, nuclear factor kappa-B; NO nitric oxide; TBARS, thiobarbituric acid reactive substances; TNF-α, tumor factor necrosis alpha; TNBS, 2,4,6 trinitrobenzenesulfonic acid; TLR, toll-like receptor.

**Table 3:** Summary of probiotic effects on IBD in human studies.

Reference	Subjects	Probiotic Strains/Treatment	Time	Main Outcome	Adverse Event/Adverse Effects
Tamaki et al., 2016 [62]	56 with mild to moderate UC	*B. longum* 536	8 weeks	Probiotics administration improved clinical symptoms in the patients with mild to moderately active UC	-
Yoshimatsu et al., 2015 [63]	60 outpatients with UC in remission	*S. faecalis*, *C. butyricum* and *B. mesentericus*	12 months	Probiotic may be effective for maintaining clinical remission in patients with UC	-
Krag et al., 2013 [64]	74 patients with a mild-to-moderate UC	*L. plantarum* 299v	8 weeks	Probiotic supplementation was safe, well tolerated, palatable, and able to reduce disease index scores in patients with mild-to-moderate UC	-
Petersen et al., 2014 [71]	100 patients with UC	*E. coli* Nissle 1917	7 weeks	There is no benefit in the use of *E. coli* Nissle as an add-on treatment to conventional therapies for active UC	-
Fedorak et al., 2015 [72]	119 patients with CD (within 30 days of ileocolonic resection and re-anastomosis	Capsules with bifidobacteria, lactobacilli, and *S. thermophilus*	90 days	There were no differences in endoscopic recurrence, but mucosal levels of inflammatory cytokines such as IL-8, IL-1β were lower among patients who received the probiotic	-
Hevia et al., 2014 [73]	50 healthy individuals, 37 CD patients and 15 UC patients	*L. casei* subsp. *rhamnosus* GG	90 days	Levels of IgA antibodies developed against a cell-wall hydrolase from *L. casei* subsp. *rhamnosus* GG were significantly higher in the IBD group	-
Ahmed et al., 2013 [74]	8 patients with CD and 8 patients with UC	*L. acidophilus* LA-5*, L. delbrueckii* subsp*. bulgaricus* LBY-27*, B. animalis* subsp. *lactis* BB-12, *S. thermophilus* STY-31 and 15 g oligofructose	1 month	There were no differences in colonic microbiota between patients with CD or UC and the spectrum a bacterium was not altered by synbiotics administration	-
Persborn et al., 2013 [75]	16 patients with chronic pouchitis and 13 individuals as a control	*L. acidophilus* Ecologic 825: *B. bifidum* (W23), *B. lactis* (W51), *B. lactis* (W52), *L. acidophilus* (W22), *L. casei* (W56), *L. paracasei* (W20), *L. plantarum* (W62), *L. salivarius* (W24) and *L. lactis* (W19)	8 weeks	Probiotics restored the mucosal barrier to *E. coli* in patients with pouchitis	-
Groeger et al., 2013 [76]	22 UC patients, 48 patients with chronic fatigue syndrome and 26 psoriasis patients	*B. infantis* 35,624	6–8 weeks	Probiotics administration reduced the systemic pro-inflammatory biomarkers in both gastrointestinal and non-gastrointestinal conditions	-
Bourreille et al., 2013 [77]	165 patients with CD	*S. boulardii*	52 weeks	Probiotics were well tolerated but it did not show any effect. Twenty-one AEs occurred during the treatment, these affected 17 patients, 9 in the *S. boulardii* group and 8 in placebo group	Twenty-one AEs occurred during the treatment, these affected 17 patients, 9 in the *S. boulardii* group and 8 in placebo group

AE, adverse event; CD, Crohn’s disease; UC, ulcerative colitis.
